# Resorbable bilayer membrane made of L-lactide-ε-caprolactone in guided bone regeneration: an in vivo experimental study

**DOI:** 10.1186/s40729-024-00520-7

**Published:** 2024-01-25

**Authors:** Taito Watanabe, Akira Hasuike, Shin Wakuda, Keisuke Kogure, Seiko Min, Norihisa Watanabe, Ryo Sakai, Akhilanand Chaurasia, Yoshinori Arai, Shuichi Sato

**Affiliations:** 1 Department of Periodontology, Nihon University School of Dentistry, 1-8-13 Kanda-Surugadai, Chiyoda-ku, Tokyo, 101-8310 Japan; 2 Division of Applied Oral Sciences, Nihon University Graduate School of Dentistry, Tokyo, 101-8310 Japan; 3 Dental Research Center, Nihon University School of Dentistry, Tokyo, 101-8310 Japan; 4 Department of Periodontics and Dental Hygiene, The University of Texas Health Science Center at Houston School of Dentistry, 7500 Cambridge Street, Houston, TX 77054 USA; 5 Department of Oral Medicine and Radiology, Faculty of Dental Sciences, King George’s Medical University, Chowk, 226003 India; 6 Department of Oral and Maxillofacial Radiology, Nihon University School of Dentistry, Tokyo, 101-8310 Japan

**Keywords:** Guided bone regeneration, Dental implant, PLACL, Resorbable bilayer membrane

## Abstract

**Purpose:**

Guided bone regeneration (GBR) is an accepted method in dental practice that can successfully increase the bone volume of the host at sites chosen for implant placement; however, existing GBR membranes exhibit rapid absorption and lack of adequate space maintenance capabilities. We aimed to compare the effectiveness of a newly developed resorbable bilayer membrane composed of poly (l-lactic acid) and poly (-caprolactone) (PLACL) with that of a collagen membrane in a rat GBR model.

**Methods:**

The rat calvaria was used as an experimental model, in which a plastic cylinder was placed. We operated on 40 male Fisher rats and subsequently performed micro-computed tomography and histomorphometric analyses to assess bone regeneration.

**Results:**

Significant bone regeneration was observed, which was and similar across all the experimental groups. However, after 24 weeks, the PLACL membrane demonstrated significant resilience, and sporadic partial degradation. This extended preservation of the barrier effect has great potential to facilitate optimal bone regeneration.

**Conclusions:**

The PLACL membrane is a promising alternative to GBR. By providing a durable barrier and supporting bone regeneration over an extended period, this resorbable bilayer membrane could address the limitations of the current membranes. Nevertheless, further studies and clinical trials are warranted to validate the efficacy and safety of The PLACL membrane in humans.

## Background

Owing to the clinical effectiveness of dental implants, dental implant treatments have emerged as the preferred alternative to dental prostheses that are supported by natural teeth or adjacent soft tissues in oral cavity. However, the volume of hard and soft tissues is often insufficient owing to changes in the ridge following tooth extraction [1]. Bone loss can negatively affect the success of dental implants. Therefore, new protocols are warranted to enhance the width and height of the alveolar crest. A commonly used method is guided bone regeneration (GBR), which involves the placement of mechanical barriers to eliminate certain cell types (e.g., rapidly proliferating epithelium and connective tissue), thereby promoting the growth of slow-growing cells capable of bone formation around natural teeth according to the theory of guided tissue regeneration [2–4]. GBR membranes serve as mechanical barriers and play a crucial role in the clinical success of GBR. Favorable characteristics of barrier membranes in regenerative therapy include biocompatibility, cell occlusion properties, integration with the host tissues, clinical adaptability, and space-making capacity [5]. The first material used as a mechanical barrier for GBR was a non-resorbable membrane made of polytetrafluoroethylene (PTFE), which effectively prevented the migration of epithelial cells to the regenerated site [6]. Titanium-reinforced PTFE, high-density PTFE, and titanium meshes have also been used, particularly for large defects [7]. However, these non-resorbable membranes have certain disadvantages including the need for a second surgical intervention to remove the membrane and the risk of infection due to membrane exposure [8].

Resorbable membranes offer the advantage of being naturally resorbed by the body, eliminating the need for second-stage removal surgery [8]. Although resorbable membranes should ideally support new bone formation and maturation for at least 6 months, the resorption pace of resorbable membranes varies among products and is clinically unpredictable, which greatly affects the bone growth even though these membranes should ideally promote new bone formation and maturation for at least 6 months [9, 10]. The most commonly used natural polymers for resorbable barrier membranes in GBR procedures are type I and type III collagen derived from the porcine dermis and bovine tendons [11]. Owen et al. [12] assessed the resorption rates of non-crosslinked collagen membranes in the alveolar bones of 12 adult dogs. They concluded that all the membranes had severe to unidentifiable degradation at 3 months, and all the membranes showed severe to completely absent degradation at 4 months. Although crosslinked membranes have longer degradation times, higher exposure rates have been reported for these membranes [13, 14]. Furthermore, most collagen membranes are weak under wet conditions and lose their space-maintaining ability under humid conditions. Moreover, there is a potential risk of unknown pathogens being associated with animal-derived collagen [15].

With synthetic biodegradable barrier membranes, the rate of absorption can be finely tuned through modification of their crystallinity, molecular weight, and hydrophobicity, resulting in an ideal new bone formation and maturation [16]. These materials include biodegradable polyesters, such as polylactic acid, polyglycolic acid, polycaprolactone, and their copolymers, polylactic-*co*-glycolic acid and polylactic-*co*-caprolactone acid [17, 18]. Since improving mechanical properties using only biodegradable polyesters is challenging, a combination of layers with different porosities has been proposed. Although numerous membranes have been developed, an ideal barrier membrane has yet to be identified.

**Fig. 1:**
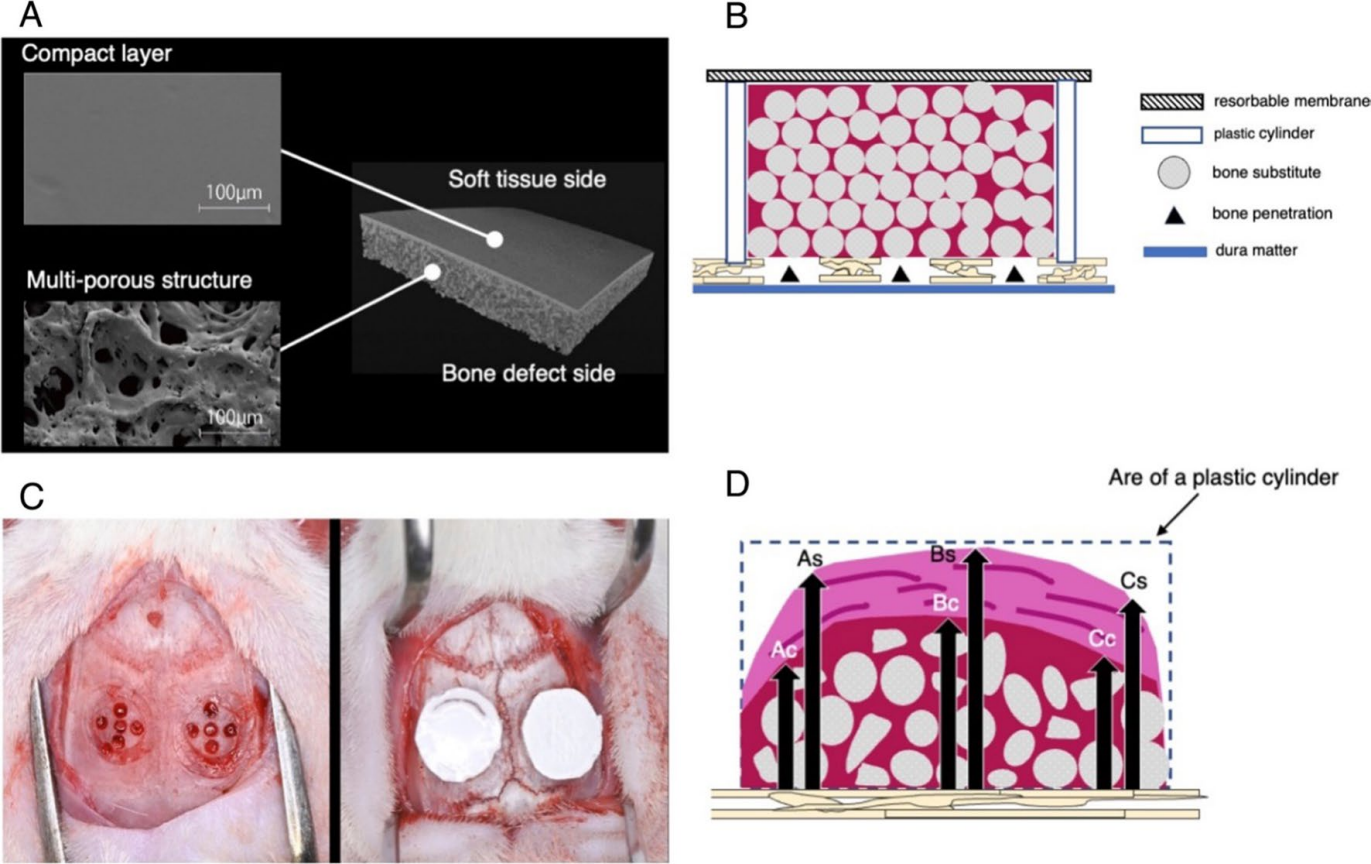
Schematic images of animal models: **A** microscopic images of both the inner and outer surfaces of poly (l-lactic acid) and poly (-caprolactone) (PLACL) provided by GC, Tokyo, Japan. **B** Each plastic cylinder is filled with the assigned bone graft and covered with the designated mechanical barriers. **C** Two surgical sites are set on each of the animal’s calvaria. **D** Quantitative measurements include the percentage of newly generated bone areas and the percentage of remaining bone substitute particles, as well as the heights of the total, calcified, and non-calcified tissues

A new resorbable bilayer membrane for GBR composed of poly (l-lactic acid) and poly (-caprolactone) (PLACL membrane) was recently developed [19]. Complete biodegradation of poly (-caprolactone) takes several months to several years, depending on the degree of crystallinity, molecular weight, and degradation conditions, such as temperature and the presence of enzymes [20]. These modifications result in a more durable and biocompatible membrane for GBR. This newly developed PLACL membrane consisted of a bilayer: a compact layer and a multiporous layer (Fig. 1A). The compact layer on the soft tissue side prevents fibroblasts from entering the bone defect site, whereas the multiporous layer on the bone defect side promotes the differentiation of undifferentiated cells into osteoblasts and allows for flexible operability owing to its multiporous structure. Abe et al. [19] immersed PLACL membranes in phosphate-buffered saline at 37 °C and studied the same at 2, 6, 12, 26, 39, and 52 weeks duration. They demonstrated that only 55% of the PLACL membranes were resorbed after 26 weeks in an in vitro setting. Thus, the PLACL membranes act as a barrier for a longer duration than other resorbable membrane products. However, histological assessments are still lacking, especially for vertical bone augmentation.

Since multiple determinants influence the success of the GBR procedure, assessment using a preclinical model is considered challenging [21]. We developed a preclinical rat GBR model that uses plastic caps placed on the calvarial defects [22]. This model is well-standardized and normalized and permits sequential radiographic examination. We previously used this model to examine the bone regenerative effects of growth factors [23], bone substitutes [24], hormones [25], and titanium membranes [26]. In the present study, we aimed to evaluate the effects of a PLACL membrane (PLACL, Cytrans Elashield®, GC, Tokyo, Japan) on bone regeneration by micro-computed tomography (micro-CT) and correlated the findings with the histological findings in our rat preclinical GBR model.

## Methods

### Experimental groups and surgical procedures

Animal experiments were approved by the Animal Experimentation Committee of Nihon University School of Dentistry (AP21D009). All applicable international, national, and/or institutional guidelines for the care and use of animals were followed.

The sample size for the study was determined using a rigorous power analysis conducted using G*Power software v. 3.1 (University of Dusseldorf, Dusseldorf, Germany). The analysis utilized an alpha level of 0.05 and a statistical power of 95%. Data from a previous study using the same animal model [26] were collected to determine the appropriate sample size. This previous study compared the percentages of newly formed tissues using two different barriers and demonstrated a statistically significant difference. The results indicated percentages of 40 ± 4.5% and 31.5 ± 4.7% for the respective barriers. Minimizing the use of animals was prioritized to a significant extent from an animal welfare perspective. Forty surgical sites were included in the study, involving 9-week-old male Fisher rats weighing 250–300 g.

The animals were acclimatized for 1 week and housed in pairs in standard cages in a controlled environment in terms of temperature, humidity, light/dark cycle, and diet. Surgery was performed under general anesthesia, initially using 4% isoflurane inhalation for 2 min, followed by an intraperitoneal injection of a mixture of dexmedetomidine hydrochloride (0.15 mg/kg), midazolam (2.0 mg/kg), and butorphanol tartrate (2.5 mg/kg). Local anesthesia was achieved with a 0.5 ml solution of 2% lidocaine with 1:80,000 epinephrine dilution to control pain and bleeding.

All the surgical procedures were performed by an experienced surgeon (N.W.). The surgical procedure involved shaving and disinfecting the area between the eyes and the posterior end of the skull with 70% ethanol. A 6.0-cm midline incision was made, and a mucoperiosteal flap was elevated to expose the cranial vertex. Bilateral circular grooves, 5 mm in diameter, were created at the center of each parietal bone using a trephine bur. Within the experimental site, five small holes (diameter: 0.5 mm) were drilled inside the circular grooves without penetrating the inner dura of the cranial bone to induce bleeding from the marrow space, thereby allowing the infusion of the bone graft materials with blood. Plastic cylinders measuring 5.0 and 3 mm in diameter and height, respectively were tightly fixed into the circular grooves on the denuded bone.

Surgical sites were randomly categorized into four distinct groups based on the type of bone graft and mechanical barriers employed. To establish a robust basis for comparison with PLACL, a widely acknowledged natural collagen membrane (COL, Bio-Gide®, Geistlich-Pharma, Wolhusen, Switzerland) was selected as the control membrane owing to its well-established reputation as the preferred absorbable membrane in clinical practice for over two decades. Considering the pivotal role of bone grafts in providing a structural scaffold for clot development, maturation, and remodeling, which is essential for supporting bone formation in osseous defects, our study incorporated two types of bone graft materials: carbonate apatite (CO3AP, Cytrans®, GC, Tokyo, Japan) and deproteinized bovine bone mineral (DBBM, Bio-Oss®, Geistlich-Pharma, Wolhusen, Switzerland), both utilized in conjunction with membranes. The four treatment combinations are as follows: (1) CO3AP + PLACL; (2) CO3AP + COL; (3) DBBM + PLACL; and (4) DBBM + COL.

Each plastic cylinder was filled with the assigned bone graft and aligned with the circular grooves. The cylinders were then covered with the designated mechanical barriers (Fig. 1B). Two surgical sites were identified in the calvaria of each animal (Fig. 1C). Finally, the mucoperiosteal flaps were repositioned using resorbable interrupted sutures (VSORB 4-0, Washiesu Medical, Tokyo, Japan). The day of surgery was designated as Day 0.

### Outcome assessments

#### Micro-CT analysis

Mineral volume at the surgical sites was assessed using an in vivo micro-CT system (R_mCT2 system; Rigaku, Tokyo, Japan) without euthanizing the rats. The rats were anesthetized with an oxygen–isoflurane mixture administered via a facemask and positioned on an imaging stage. The exposure parameter was set to 90 kV. The region of interest for micro-CT assessment was the circular grooves (diameter: 5.0 mm, height: 3.0 mm) on the calvaria. Images were reconstructed using the i-View software (i-View Image Center, Tokyo, Japan) on a personal computer. The bone volume within the plastic caps in the three-dimensional voxel images was analyzed using the same software. The enhanced volume was calculated by subtracting the volume on Day 0 from that at each follow-up. Micro-CT assessments were performed by a single, blinded examiner (T.W.) under the supervision of an experienced examiner (Y.A.).

#### Histomorphometric analysis

After 24 weeks postoperatively, all the rats were euthanized by administering excess CO_2_ gas after the last micro-CT scan. The calvarial bone defects were resected following skin dissection. Bone segments containing the cylinders were surgically removed and fixed in 10% neutral-buffered formalin. After fixation, the bone specimens were decalcified in 5% formic acid for 14 days and embedded in 2-hydroxyethyl methacrylate using routine methods. Each embedded bone specimen was cut into 5-μm sections (HistoCore AUTOCUT; Leica, Wetzlar German) and air-dried at 50 ℃. The samples were stained with hematoxylin and eosin (HE).

Histomorphometric analyses were performed using histological sections obtained from within the plastic cylinders under a light microscope and an image analyzer computer system using FIJI (ImageJ 1.50b or 1.52i; National Institutes of Health, Bethesda, MD, USA). We assessed osteogenesis (bone formation) at the experimental GBR sites.

Quantitative measurements were performed on each HE-stained histological section. These measurements included the percentage of newly generated bone area; the percentage of remaining bone substitute particles; and the heights of the total, tissue, and non-calcified tissues (Fig. 1D). Each height measurement was calculated as the average of three different locations: the horizontal center of the plastic cylinder and both parts located 1 mm away from the center. The measurements for histomorphometric assessment were performed by an experienced and blinded examiner (S.W.).

### Statistical analysis

The means and standard deviations (SD) of each variable were calculated and analyzed using EZR (Saitama Medical Center, Jichi Medical University, Saitama, Japan), a graphical user interface for R 2.13.0 (R Foundation for Statistical Computing, Vienna, Austria). To assess the statistical significance of the bone volumes obtained from the micro-CT images and the percentages of residual bone substitutes in HE stain, a Kruskal–Wallis test with a Steel post-hoc test was performed. Statistical significance was set at *p* < 0.05.

## Results

### Postoperative visual observation

After the surgical procedures, all the rats displayed excellent tolerance and made a swift and uneventful recovery. Notably, no signs of wound dehiscence, graft migration, infection, or other complications were observed.

### Micro-CT analysis

**Fig. 2:**
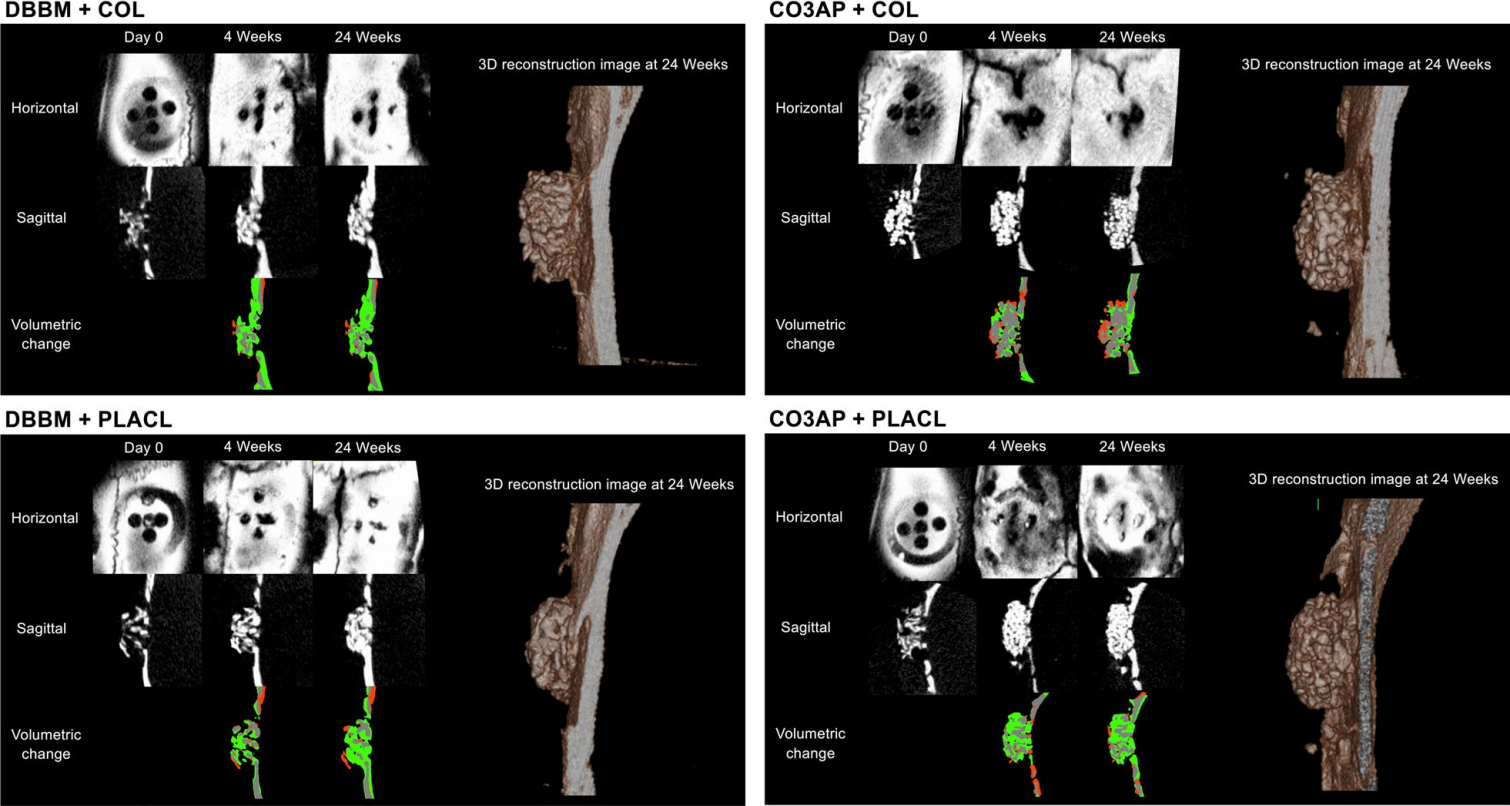
Micro-computed tomography analysis in all four groups. The green color in the volumetric change indicates newly added voxels; the red color in the volumetric change indicates diminished voxels

**Fig. 3:**
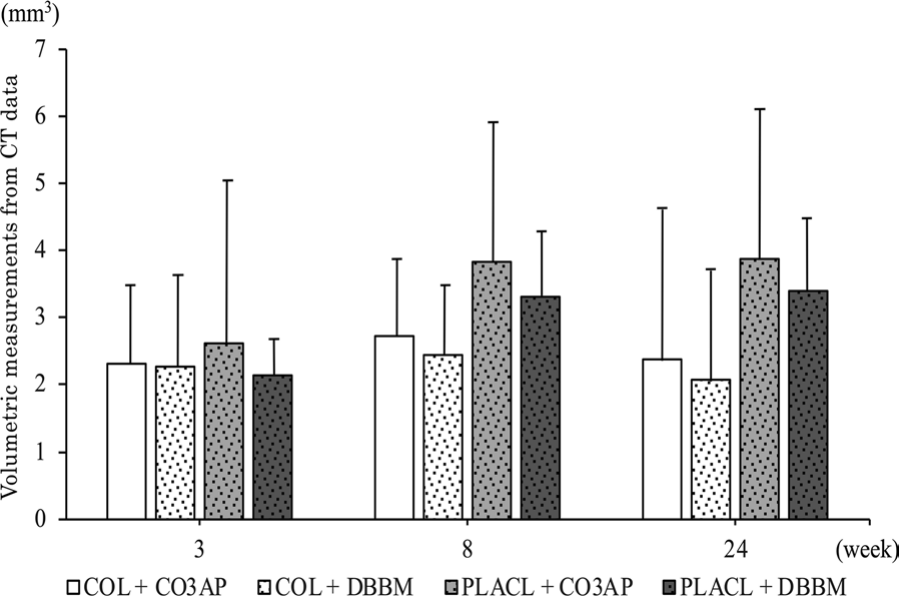
Volumetric measurements from the micro-computed tomography data (mm^3^). Values are presented as mean (standard deviation). There were no statistical differences between treatment groups (*p* > 0.05)

Figure 2 shows the micro-CT images of the representative surgical sites from each group, offering enhanced visualization. The three-dimensional micro-CT images revealed a gradual improvement in the radiopaque contrast within the cylinders in all four groups. In specific, the gap between the particles appeared to be partially filled with radiopacity in all four groups in the sagittal images captured at the 24-week milestone. Notably, throughout the observation period, the distinct characteristics of each DBBM particle were distinguishable. This was contrary to the aggregated appearance of CO3AP, which appeared as a conglomeration of multiple particles. However, these differences in particle appearance did not have any impact on the voxel-based volumetric bone analysis. Quantitative volumetric analysis revealed a progressive increase in the bone volume within the cylinders in all four groups. The total voxel count increased in a time-dependent manner until 8 weeks and reached a state of equilibrium thereafter. The observed differences among the four groups were not statistically significant (Fig. 3).

### Histomorphometric analysis

**Fig. 4:**
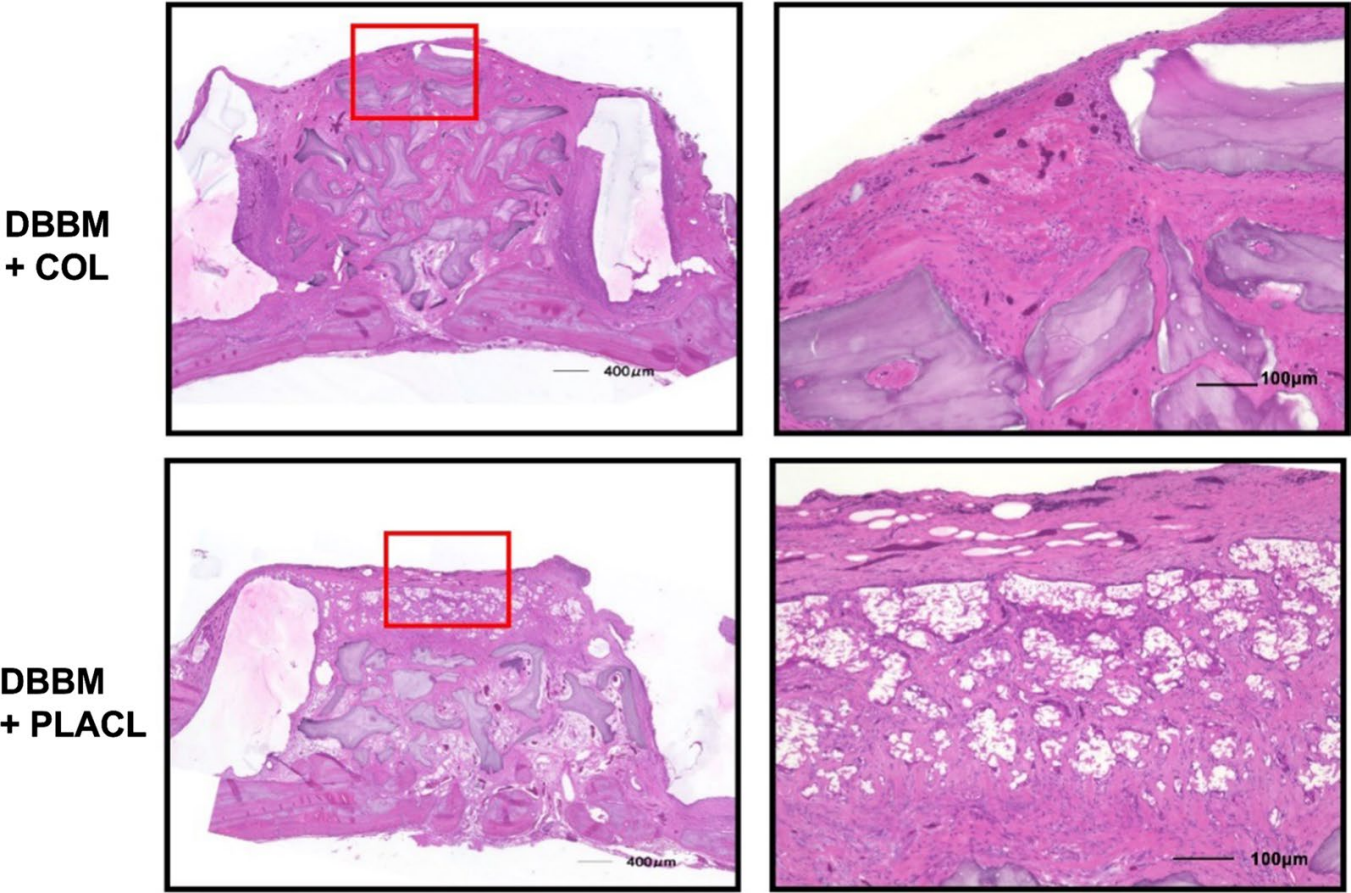
Representative histological specimens of the deproteinized bovine bone mineral (DBBM) groups at week 24. Left: lower magnification; right: higher magnification

**Fig. 5:**
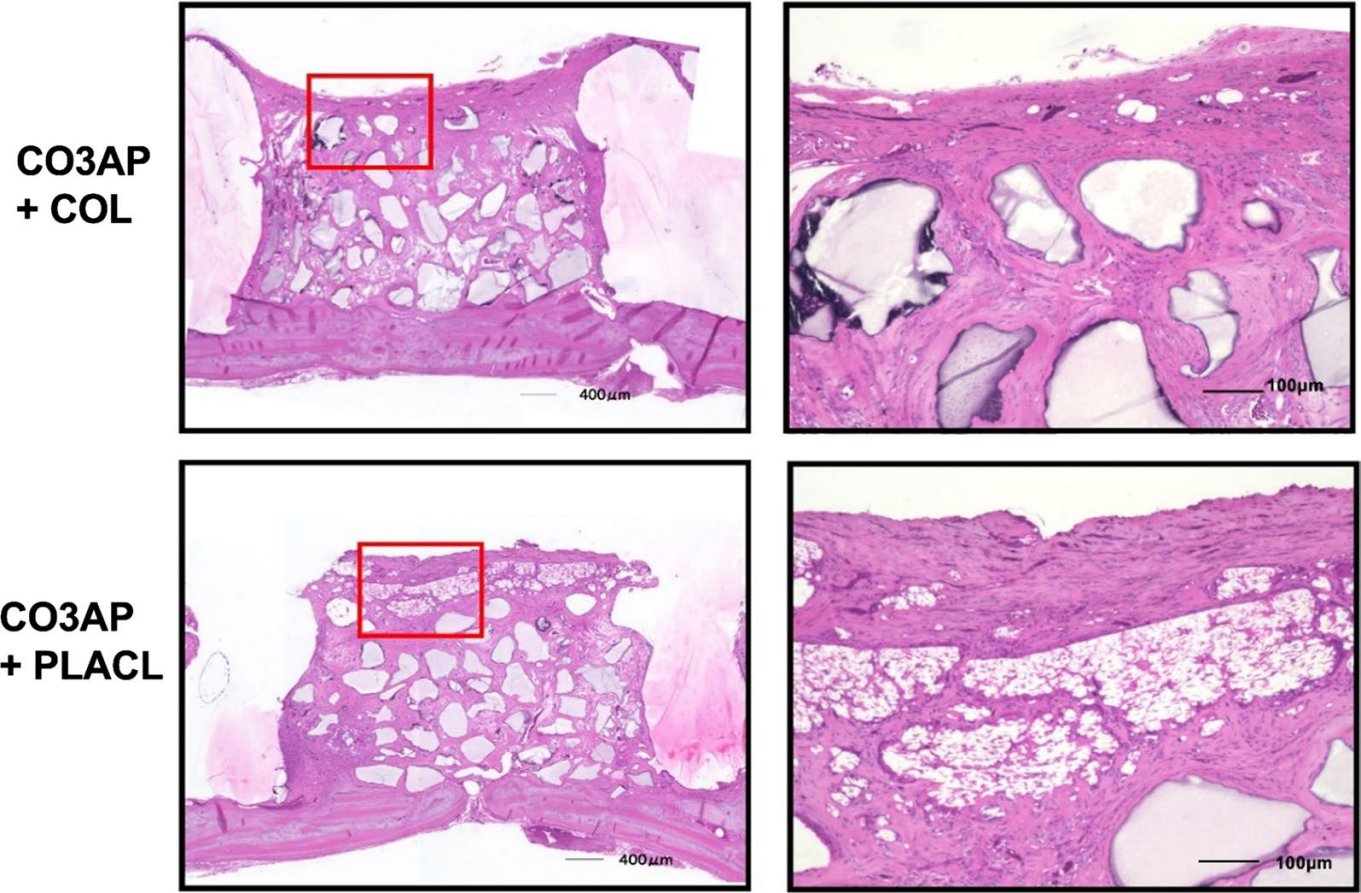
Representative histological specimens of the CO3AP groups at week 24. Left: lower magnification; right: higher magnification

Figure 4 shows the histology of the rats treated with DBBM. At 24 weeks postoperatively, both the groups treated with COL and PLACL exhibited significant bone augmentation, as observed in the images. The bone-substituted particles were surrounded by newly formed bone tissue without any signs of inflammation or heterogenization. The percentage of residual bone substitutes was similar for both types of bone substitutes: 31.4% for COL and 26.4% for PLACL. Figure 5 shows the histology of the samples treated with CO3AP at 24 weeks postoperatively. The CO3AP-treated rats exhibited a trend similar to that of the DBBM-treated rats. The analysis revealed no significant differences in the percentage of residual bone substitutes between the CO3AP + COL and CO3AP + PLACL groups (29.1% vs. 33.3%).

**Fig. 6:**
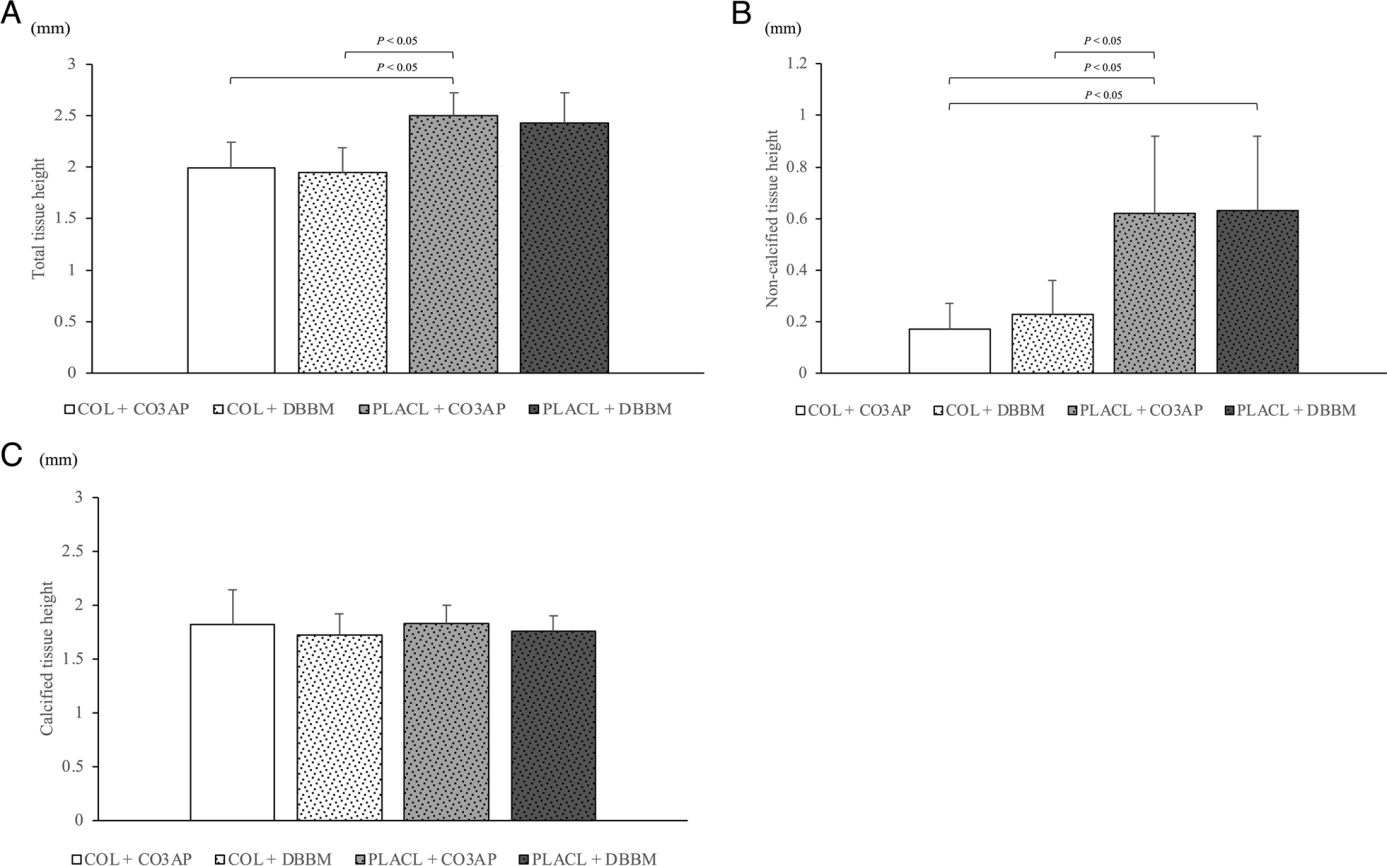
Measurements of the tissue height: **A** height of total tissue. **B** Height of non-calcified tissue. **c** Height of calcified tissue

The results of tissue height measurements are illustrated in Fig. 6. The total tissue volume exhibited a significant increase in the CO3AP + PLACL group (2.43 mm) compared to that in the DBBM + COL (1.95 mm) and CO3AP + COL (1.99 mm) groups (Fig. 6A). This disparity was primarily driven by the heightened non-calcified tissue. In specific, the non-calcified tissue volume displayed a substantial increase in the CO3AP + PLACL group (0.62 mm) in contrast to that in the DBBM + COL (0.23 mm) and CO3AP + COL (0.17 mm) groups (Fig. 6B). A significant difference was also shown when comparing non-calcified tissue between the DBBM + PLACL (0.63 mm) and CO3AP + COL (0.17 mm) groups (Fig. 6B). Conversely, no noteworthy differences were identified in the height of the calcified tissue among the four groups (Fig. 6C).

## Discussion

In the present study, we conducted a comprehensive analysis to evaluate and compare the effects of PLACL membranes on bone regeneration in a rat model of calvarial vertical GBR with COL membranes. Three-dimensional micro-CT analysis revealed a gradual improvement in the radiopaque contrast within the cylinders in all the groups. The particles were distinguishable, and a progressive increase was observed in bone volume within the cylinders over time. Histomorphometric analysis at 24 weeks also revealed significant bone augmentation in both the PLACL and COL groups. In 24 weeks, the PLACL groups demonstrated more total tissue volume and thicker non-calcified tissue than those of the COL groups. These results were independent of the type of bone graft material used. Based on these results, PLACL membranes has been successfully demonstrated to overcome the challenges related to the infiltration of epithelial cells into the bone, which can impede proper bone formation.

**Fig. 7:**
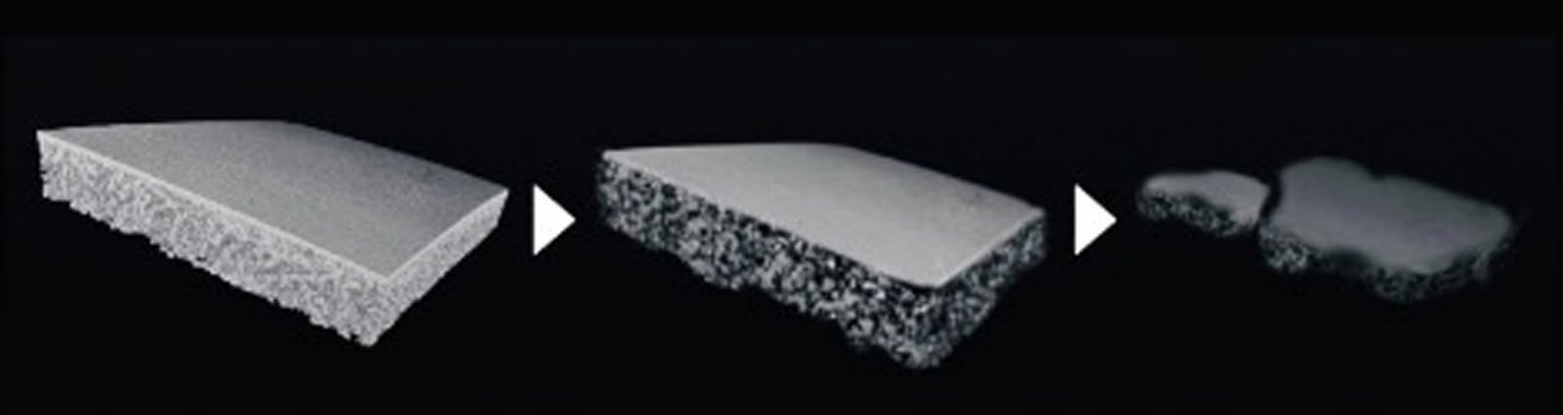
Schematic images of poly (l-lactic acid) and poly (-caprolactone) (PLACL) degradation provided by GC, Tokyo, Japan

A previous in vitro study demonstrated resorption of 55% of the PLACL membranes after 26 weeks [19]. Based on these findings, we evaluated the dynamic structural changes in bone regeneration in our rat model using micro-CT scans for 24 weeks. This imaging technique allowed us to visualize and quantify important parameters, such as bone volume and morphology. Our results revealed a gradual increase in the bone volume over 8 weeks in the two groups utilizing the PLACL and collagen membranes, indicating successful bone regeneration. Hence, based on our findings, we conclude that the critical period for the blocking effect of the GBR membrane is approximately 8 weeks. This crucial timeframe signifies the optimal duration during which the membrane effectively prevents unwanted tissue ingrowth and facilitates proper tissue regeneration. Our histological analysis revealed partially degraded PLACL membranes after a 24-week follow-up period. The 24-week follow-up period represents a remarkable achievement in the field of tissue regeneration research, particularly when considering its extended duration for studies performed on rats. This extended timeframe allows for a comprehensive evaluation of the long-term effects of the treatment, providing valuable insights into the sustained efficacy and stability of the regenerative process. Our histological findings correspond with those of previous in vitro studies concerning material degradation and support a prolonged barrier effect during GBR protocols [20, 27]. Figure 7 illustrates the time-dependent degradation of the PLACL material using schematic images. Notably, even at 24 weeks post-transplantation, the PLACL membrane showed significant resilience. This extended preservation of the barrier effect shows great promise in facilitating optimal bone regeneration. Sporadic instances of partial degradation were noted in both groups with the PLACL membrane, accompanied by sporadic air bubble-like lesions. While these occurrences are presumably attributed to the variations in the absorption tendencies based on the bilayer structure of the PLACL membrane, the impact of this phenomenon remains elusive and warrants further investigation.

The current study did not specifically confirm the presence of inflammation above and beneath the PLACL membranes. This characteristic is primarily attributed to the exceptional biocompatibility of this PLACL membrane. In a previous study by Abe et al. [28], bacteria were directly seeded and collected onto the compact layer of the PLACL membrane. *Porphyromonas gingivalis*, *Streptococcus mutans*, and multispecies bacteria were seeded on the compact layer of a PLACL membrane, and bacterial adhesion was assessed at 6, 24, and 72 h. Bacterial penetration was also assessed using a scanning electron microscope at 24 and 72 h. Bacteria adhered to all the membranes after only after 6 h of incubation. The PLACL membrane could block bacterial penetration, and no bacterial cells were observed in the structure. In contrast, bacteria penetrated both control membranes and were observed at depths of up to 80 μm following 72 h of incubation. Significantly fewer adherent bacteria were observed on the PLACL bilayer membrane than on the control membranes throughout the experimental period. Moreover, the PLACL membrane effectively blocked bacterial penetration, as no bacterial cells were detected within its structure. The unique structure of the PLACL membrane contributes to its barrier function against both bacteria and epithelial cells.

In the current study, two different types of particulate bone substitutes were used in cylinders covered with two distinct barrier membranes: DBBM and CO3AP. DBBM is a widely used bone graft material, especially in regions where the use of human-donated grafts is prohibited. Although xenografts are the initial standard replacement materials, such as autografts and allografts, they are generally not resorbed over time. Recently, a CO3AP bone substitute has been developed as an alternative to allografts and xenografts. Commercially available CO3AP particles are chemically pure and are produced through a dissolution–precipitation reaction in a water-based solution using a calcite block instead of through a sintering process. This innovative manufacturing method ensures that the CO3AP particles retain their biodegradability. This result aligns closely with our previous report, which also utilized the same model and had a follow-up period of 12 weeks [25]. However, it is important to note that our histological analysis conducted over 24 weeks revealed no resorption of the DBBM or CO3AP particles.

The findings of present study have significant implications for clinical practice, particularly in addressing issues associated with the use of animal- or human-derived materials. The combination of the synthesized absorbable materials, PLACL membranes, and CO3AP bone substitutes is a promising solution. The potential practical application of the presented options provides positive prospects for patients needing such medical interventions.

A limitation of this study is the utilization of an animal model, specifically, the rat calvaria, which may not accurately reflect the behavior and response of human jawbones. Although the current study demonstrated partial resorption of the membrane over 24 weeks, whether similar results would be observed in humans remains uncertain. In addition, observations made during the 24 weeks revealed limited resorption on the PLACL membranes and no resorption on CO3AP bone substitutes. Consequently, conducting further long-term observations is crucial for gaining a more comprehensive understanding of the long-term behavior and potential resorption patterns of these materials. By extending the observation period, researchers could collect more robust data and insights, ultimately providing a more accurate assessment of the clinical viability and effectiveness of the PLACL membranes and CO3AP bone substitutes.

## Conclusions

The barrier effect was maintained by the PLACL membranes in the rat calvarial vertical GBR model for a prolonged duration. These results demonstrate the potential use of the PLACL membrane as a robust, resorbable barrier that promotes bone repair.

## Data Availability

Not applicable.

## Abbreviations

CT: Computed tomography
COL: Collagen
CO3AP: Carbonate apatite
DBBM: Deproteinized bovine bone mineral
GBR: Guided bone regeneration
HE: Hematoxylin and eosin
PLACL: Poly (l-lactic acid) and poly (-caprolactone)
PTFE: Polytetrafluoroethylene
SD: Standard deviations
